# Deciphering Crosstalk Circuits in Non-small Cell Lung Cancers with an Increasing Interval Length of Low Dose CT Screening

**DOI:** 10.1016/j.ebiom.2015.07.026

**Published:** 2015-07-21

**Authors:** Rafael Rosell, Niki Karachaliou, Imane Chaib, Sara Pilotto, Emilio Bria, Juan Luis Fernández-Martínez, Jose Luis Ramirez

**Affiliations:** a Catalan Institute of Oncology and Germans Trias i Pujol Research Institute, Badalona, Barcelona, Spain; b Quirón-Dexeus Hospital, Barcelona, Spain; c Department of Medicine, University of Verona, Azienda Ospedaliera Universitaria Integrata, Verona, Italy; d Department of Mathematics, University of Oviedo, Oviedo, Spain

There is growing evidence of an accumulation of mutations and mechanisms of resistance to targeted therapies in cancer. Non-small-cell lung cancer (NSCLC) is still an untreatable disease and is the leading cause of cancer related deaths worldwide (Gridelli et al., 2015). Various strategies have been proposed to increase the survival of NSCLC patients, including early diagnosis (Gridelli et al., 2015). Indeed, 5-year survival rates can reach 70% with surgical resection in stage IA disease. No benefit has been shown for adjuvant chemotherapy in stage IA. However, most patients are diagnosed at later stages (at initial diagnosis 55% of patients have metastatic disease) (Gridelli et al., 2015). NSCLC gene signatures have uncovered typical protein-coding gene mutations as well as subsets of microRNAs (miRNAs) that are aberrantly expressed (Rosell & Karachaliou, 2015). The main challenge faced by physicians doing CT screening for lung cancer is that about half of the individuals screened have one or more pulmonary nodules, but only a small percent of these individuals have lung cancer (Gridelli et al., 2015). In the Multicenter Italian Lung Detection (MILD) trial the plasma miRNA signature was predictive of malignancy risk and was able to distinguish lung cancers from the majority of benign pulmonary nodules detected by low-dose CT (Sozzi et al., 2014). Interestingly, two tumor-suppressive miRNAs, let-7 and miR-34, are the most common altered miRNAs in NSCLC tumor tissue The reduction in let-7 and miR-34 expression is relevant in the NSCLC oncogenic phenotype and is involved in the maintenance of oncogene addiction, via RAS, BCL2, MET or MYC. Systemic nanodelivery of miR-34 and let-7 suppresses tumor growth. This combinatorial miRNA approach engages numerous components of tumor cell-addictive pathways and could eventually result in a novel therapeutic approach (Kasinski et al., 2015). Small nodules (those with a volume < 100 mm (Sozzi et al., 2014) or a diameter < 5 mm) are not predictive for lung cancer. Immediate diagnosis evaluation is warranted for large nodules (> 300 mm (Sozzi et al., 2014) or a diameter > 10 mm). Based on the NELSON trial, volume doubling time assessment is also recommended for intermediate size nodules (Horeweg et al., 2014). In addition, most patients with lung adenocarcinoma with sub-1-cm lung tumors do not present lymph node metastases or recur with metastatic disease. This is likely due to the inability of cells to disseminate, however, unfortunately, even patients who have potentially curative surgery often relapse to the presence of metastasis below detection level at the time of diagnosis. Molecular mediators of NSCLC metastasis have been reported by Hu et al. who defined gene signatures that stratify the recurrence-free-survival (Hu et al., 2015). The authors of the MILD study (Sozzi et al., 2014) have now identified through a multi-gene signature that tumors identified by CT screening can be distinguished between indolent and aggressive tumors, with different free-recurrence survivals at 5 years in stage I NSCLC patients. Their results also point out that more virulent tumors are those identified in the later years of follow-up and the gene signature indicates a hyper-activation of the PI3K–PTEN–AKT pathway with up-regulation of ITGB1 and inhibition of FOXO1A expression (Hu et al., 2015). The phosphatidylinositol 3-kinase (PI3K) is a universal tumor driver that integrates growth factor signaling with downstream circuitries of cell proliferation. Hu et al. have found that developing lung cancer between 3 and 5 years of CT screening more frequently involves increased gene expression of β Integrins (ITGB1), as shown in Fig. 5, where ITGB1 directly interacts with Integrin-linked kinase (ILK) and phosphorylates AKT in a PI3K-dependent manner (Hu et al., 2015). NSCLC is frequently associated with epidermal growth factor receptor (EGFR) over expression, gene amplification and acquired gain of function mutations. EGFR signaling in NSCLC promotes tumor growth, survival and metastasis (Gridelli et al., 2015). EGFR and other growth factors activate the crosstalk between signaling pathways (Rosell et al., 2013), including ILK/AKT, extracellular signal regulated kinase ½ (ERK1/2), signal transducers and activators of transcription 3 (STAT3) and NOTCH1 and HES1. Blockade of PI3K or ILK signaling with selective compounds could inhibit the phosphorylation of AKT and glycogen synthase kinase 3β, suppress STAT3 and ERK1/2 activation and also decrease NOTCH1 and HES1 expression. Hypoxia is responsible for activating ILK–AKT–Y box-binding protein 1 (YB-1), leading to the inhibition of FOXO3A and activation of epithelial–mesenchymal transition markers (ZEB1, Twist) and expression of biomarkers associated with cancer stem cell signaling pathways (Chou et al., 2015). Fig. 5 (Hu et al., 2015) epitomizes the crosstalk between signaling pathways that could be traced with biomarkers in future studies and could serve as theranostic markers for defining the free recurrence interval and adequate targeted therapy. A major caveat is that single targeted therapy can promote tumor adaptation and paradoxically promote tumor invasion and metastases (Arasada et al., 2014). In summary, Hu et al. (2015) highlight the need for PCR array analysis of important components of signaling pathways that could provide prompt answers on tumor behavior and serve to customize combinatorial therapies.
